# ANGPTL3 as therapeutic target

**DOI:** 10.1097/MOL.0000000000000789

**Published:** 2021-10-08

**Authors:** Sander Kersten

**Affiliations:** Nutrition, Metabolism and Genomics Group, Division of Human Nutrition and Health, Wageningen University, the Netherlands

**Keywords:** ANGPTL3, atherosclerosis, LDL-C, lipoprotein lipase, triglycerides

## Abstract

**Purpose of review:**

Elevated LDL-C and triglycerides are important risk factors for the development of atherosclerotic cardiovascular disease. Although effective therapies for lipid lowering exist, many people do not reach their treatment targets. In the last two decades, ANGPTL3 has emerged as a novel therapeutic target for lowering plasma LDL-C and triglycerides. Here, an overview of the recent literature on ANGPTL3 is provided, focusing on the therapeutic benefits of inactivation of ANGPTL3 via monoclonal antibodies, antisense oligonucleotides, and other more nascent approaches. In addition, the potential mechanisms by which ANGPTL3 inactivation lowers plasma LDL-C are discussed.

**Recent findings:**

ANGPTL3 is a factor secreted by the liver that inhibits lipoprotein lipase and other lipases via the formation of a complex with the related protein ANGPTL8. Large-scale genetic studies in humans have shown that carriers of loss-of-function variants in ANGPTL3 have lower plasma LDL-C and triglyceride levels, and are at reduced risk of atherosclerotic cardiovascular disease. Clinical studies in patients with different forms of dyslipidemia have demonstrated that inactivation of ANGPTL3 using monoclonal antibodies or antisense oligonucleotides markedly lowers plasma LDL-C and triglyceride levels.

**Summary:**

Anti-ANGPTL3 therapies hold considerable promise for reducing plasma LDL-C and triglycerides in selected patient groups.

## INTRODUCTION

Elevated plasma levels of LDL-C are a well known risk factors for atherosclerotic cardiovascular disease (ASCVD). The strong causal association between plasma LDL-C and ASCVD forms the basis for the use of aggressive LDL-lowering therapies in individuals at high risk of ASCVD [1]. Effective therapies are available for lowering plasma cholesterol in most people, including statins, selective cholesterol absorption inhibitors, and PCSK9 inhibitors. In contrast, the therapeutic options are more limited for individuals with homozygous familial hypercholesterolemia (HoFH). For these patients, as well as for hypercholesterolemic individuals who are largely refractory to the above treatments, there is a continued need for new treatments that can lower plasma LDL-C levels.

Apart from LDL-C, there is mounting evidence from human genetic studies that elevated plasma triglycerides are also a risk factor for ASCVD [2,3]. The positive association between triglycerides and ASCVD has been attributed to the proatherogenic effects of partially metabolized triglyceride-rich lipoproteins and their remnants. An overview of the role of triglyceride-rich lipoproteins in ASCVD can be found elsewhere [4]. The interest in finding new lipid-lowering medication with additional clinical benefit has provided the basis for continued investigations into how plasma lipoprotein metabolism is regulated, how lipid metabolism goes awry in dyslipidemia, and how certain genes and proteins can be targeted to improve lipid levels and reduce ASCVD risk. Considerable attention has been focused on the enzyme lipoprotein lipase (LPL) [5]. LPL catalyzes the hydrolysis of plasma triglycerides and is rate limiting for triglyceride uptake into muscle, heart, and adipose tissue. Due to its importance in plasma lipid metabolism, the activity of LPL in different tissues is carefully regulated to be able to cater lipid uptake to local lipid demand [5]. Several proteins are involved in the regulation of LPL activity. These proteins can be divided into the apolipoproteins C1, C2, C3, E and A5, and the angiopoietin-like proteins ANGPTL3, ANGPTL4, and ANGPTL8. With the approval of monoclonal antibodies against ANGPTL3 by the Food and Drug Administration (FDA) and the European Medicines Agency as an add-on treatment for patients with HoFH, ANGPTL3 has gained widespread recognition as a therapeutic target. Here, I will review the recent literature on ANGPTL3 and discuss how ANGPTL3 can be targeted to lower lipid levels and reduce risk of ASCVD. 

**Box 1:**
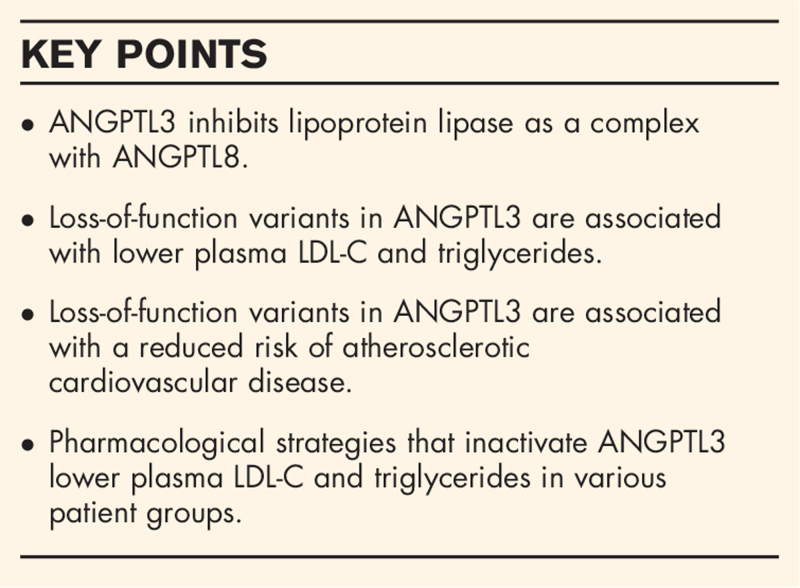
no caption available

## ROLE OF ANGPTL3 IN LIPOPROTEIN METABOLISM

ANGPTL3 is a 45 kDa protein that is exclusively produced in hepatocytes. Early studies in ANGPTL3-deficient and ANGPTL3-overexpressing mice showed that ANGPTL3 impairs clearance of triglyceride-rich lipoproteins and raises plasma levels of triglycerides by inhibiting the activity of LPL [6,7], and possibly hepatic lipase [8,9]. In addition, ANGPTL3 raises plasma HDL-C levels by inhibiting endothelial lipase [10]. The other two angiopoietin-like proteins have functions complementary to ANGPTL3. Unlike ANGPTL3, ANGPTL4 is produced by many cells and tissues, including liver, adipose tissue, heart, and macrophages. It is mainly known to serve as LPL inhibitor in adipose tissue during fasting [11]. ANGPTL8, which is produced in the liver and adipose tissue, is different from the other two proteins in that it is substantially smaller and is unable to inhibit LPL by itself. Instead, it cooperates with ANGPTL3 and ANGPTL4 to enhance or diminish their LPL inhibitory properties, respectively, as explained below.

**FIGURE 1:**
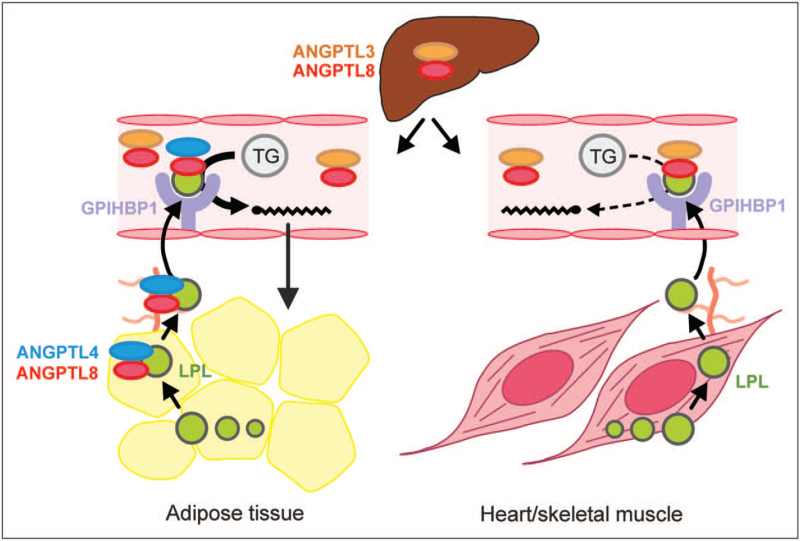
Function of ANGPTL3 in plasma triglyceride metabolism. ANGPTL3 is produced in the liver partly as a complex with ANGPTL8. This ANGPTL3/ANGPTL8 complex inhibits lipoprotein lipase (LPL) in muscle and heart in the fed state. In white adipose tissue, the inhibition of LPL by ANGPTL3/ANGPTL8 is counteracted by a complex of ANGPTL4/ANGPTL8. This mechanism ensures the preferential uptake of plasma triglycerides by adipose tissue after a meal.

Although it was for a long time believed that ANGPTL3 is a direct and independent inhibitor of LPL, it is now evident that the ability of ANGPTL3 to raise plasma triglycerides depends on ANGPTL8 (Fig. 1) [12]. Specifically, ANGPTL3 is released from liver cells partly as a complex with ANGPTL8 [13,14], which can be detected in human blood plasma [15^▪▪^]. Whereas nearly all ANGPTL8 released from hepatocytes is complexed to ANGPTL3, most of the ANGPTL3 released and present in blood plasma is in free form [15^▪▪^]. The physical association with ANGPTL8 greatly increases the affinity of ANGPTL3 for LPL and creates a very potent endocrine inhibitor of plasma triglyceride clearance in heart, skeletal muscle and brown adipose tissue [16^▪^]. In white adipose tissue, however, the inhibitory action of ANGPTL3/ANGPTL8 towards LPL is counteracted by a complex of ANGPTL4/ANGPTL8 (Fig. 1) [15^▪▪^,16^▪^,^17^^▪▪^]. In the fed state, when ANGPTL8 production is high, this mechanism ensures the preferential storage of circulating triglycerides in white adipose tissue. Whether inhibition of endothelial lipase by ANGPTL3 is enhanced by ANGPTL8 and is mediated by the ANGPTL3/ANGPTL8 complex is unknown. Interestingly, recent evidence suggests that the inhibition of LPL by ANGPTL3/ANGPTL8 is opposed by APOA5 through direct binding to the ANGPTL3/ANGPTL8 complex [18].

Human genetic studies support the role of ANGPTL3 in regulating plasma triglyceride levels. Genome-wide association studies first linked several loci near the *ANGPTL3* gene to plasma triglycerides [19]. Subsequently, exome sequencing identified *ANGPTL3* as the gene mutated in familial combined hypolipidemia, a condition characterized not only by low fasting plasma triglyceride levels but also low levels of LDL-C and HDL-C [20]. Consistent with these data, carriers of loss-of-function variants in ANGPTL3 have markedly lower levels of triglycerides (17–27%) and LDL-C (9%) [21,22]. By contrast, there only seems to be a very weak association between ANGPTL3 loss-of-function variants and plasma HDL-C levels [21,22]. The changes in plasma lipid levels in carriers vs. noncarriers of ANGPTL3 loss-of-function variants are associated with a reduction in the odds of ASCVD of about 40% [21].

## PHARMACOLOGICAL STRATEGIES FOR ANGPTL3 INACTIVATION

The liver-specific expression of ANGPTL3, its circulation in the bloodstream, and the reduction in ASCVD risk in ANGPTL3 loss-of-function carriers make ANGPTL3 a very attractive pharmacological target for lipid lowering. Several strategies, which currently are in different stages of the R&D pipeline, have been developed to inactivate ANGPTL3 and improve plasma lipid levels.

### Monoclonal antibodies

A monoclonal antibody targeting the C-terminal LPL-inhibitory domain of ANGPTL3 was developed by Regeneron. They are sold under the brand name Evkeeza but are generally referred to as Evinacumab. In preclinical studies in mice, Evinacumab increased postheparin LPL activity and reduced circulating plasma triglycerides, LDL-C, and HDL-C [23]. Similarly, Evinacumab significantly reduced plasma triglycerides, nonHDL-C, and HDL-C in dyslipidemic cynomolgus monkeys [23]. In atherosclerosis-prone APOE3L.CETP mice, the reduction in plasma cholesterol and triglycerides by Evinacumab was accompanied by an about 40% decrease in atherosclerotic lesion size [21]. Consistent with the animal data, Evinacumab significantly reduced plasma triglycerides, LDL-C, and HDL-C in phase 1 and 2 trials in patients with mixed dyslipidemia or HoFH [21,24,25]. In a recent phase 3 trial, 24 weeks of Evinacumab treatment proved to be very effective in lowering LDL-C (47%), HDL-C (30%) and triglycerides (55%) in HoFH patients [26^▪▪^]. Because of defective LDL receptors, HoFH patients are not only at greatly increased risk of ASCVD but unfortunately also do not respond sufficiently to standard cholesterol-lowering medication. The nearly 50% decrease in LDL-C levels by Evinacumab is expected to confer a substantial reduction in ASCVD risk in HoFH patients. Indeed, treatment of two teenagers with HoFH and under intense lipid-lowering therapy with Evinacumab led to marked plague regression [27]. For this reason, it is expected that Evinacumab (or other ANGPTL3-inactivating drug) will become part of the standard treatment protocol for HoFH patients and reduce the frequency of apheresis. In addition to this group, Evinacumab may be clinically useful for a much wider group of individuals at very high risk of ASCVD, including familial hypercholesterolemia heterozygotes. Supporting this notion, Evinacumab lowered plasma LDL-C by more than 50% in a phase 2 trial in patients with or without HeFH who had refractory hypercholesterolemia [28^▪▪^]. One of the disadvantages of Evinacumab is the required high frequency of injections (approximately every 4 weeks) as opposed to an injection every couple of months or years for the other therapeutic strategies described below.

### Gene silencing

In preclinical studies in various mouse models of dyslipidemia, silencing of ANGPTL3 using GalNAc-conjugated antisense oligonucleotides (ASO) significantly reduced plasma triglycerides, HDL-C, and LDL-C levels [29]. The most pronounced effects were observed in *Ldlr*-/- mice. In these mice, ANGPTL3 ASO also slowed down the progression of atherosclerosis. Mechanistic studies showed that ANGPTL3 ASO significantly enhanced clearance of triglyceride-rich lipoproteins concurrent with an increase in postheparin plasma LPL activity [29]. Similar to ANGPTL3 ASO, ANGPTL3 RNAi significantly reduced plasma triglycerides, HDL-C, and LDL-C levels in different mouse models of dyslipidemia [30].

In line with the animal data, treatment with ANGPTL3 ASO (also referred to as Vupanorsen) significantly reduced plasma ANGPTL3, triglyceride, LDL-C, and HDL-C levels in two phase 1 trials in healthy adults, with the magnitude of the decrease depending on the dose and frequency of the injections [29,31]. In a recent phase 2 trial in type 2 diabetes patients with hepatic steatosis and hypertriglyceridemia, ANGPTL3 ASO compared with placebo significantly and dose-dependently decreased plasma triglycerides to a maximum of 53%. At the highest dose, ANGPTL3 ASO also significantly reduced plasma HDL-C (24%) but not LDL-C [32^▪▪^]. Interestingly, whereas in mice ANGPTL3 ASO decreased hepatic fat content [29], this effect was not reproduced in human patients [32^▪▪^]. Similar to ANGPTL3 ASO, silencing of ANGPTL3 in healthy volunteers using ANGPTL3 RNAi (ARO-ANG3) dose-dependently reduced plasma ANGPTL3, LDL-C, and triglyceride levels to a maximum of 83, 30, and 67%, respectively [33]. Furthermore, in HeFH patients and non familial hypercholesterolemia patients with refractory hypercholesterolemia, repeated dosing with ARO-ANG3 significantly reduced mean ANGPTL3 levels between 62 and 92%, LDL-C between 23 and 37%, and triglycerides between 25 and 43% [34]. A phase 2b clinical study of ARO-ANG3 was recently initiated.

Collectively, these data show that ANGPTL3 silencing by either ASO or RNAi leads to marked reductions in plasma triglycerides, HDL-C and depending on the patient group, in LDL-C levels. It is unclear whether the observed differences in outcome of trials with ANGPTL3 monoclonal antibodies, ASO, and RNAi are because of differences in the biological mode of action or because of differences in the patient groups being studied.

### CRISPR-based gene editing

Following the seminal work of Nobel laureates Charpentier and Doudna as well as others, CRISPR-based gene editing has emerged as a promising new strategy for treating patients with atherogenic dyslipidemia. It was shown that adenoviral-mediated in-vivo based editing of ANGPTL3 significantly reduced plasma ANGPTL3, triglycerides, and cholesterol in different types of mice. Despite an efficiency of gene targeting of only 35%, plasma triglycerides and LDL-C were reduced by more than 50% in *Ldlr*-/- mice [35]. Using an alternative approach involving lipid nanoparticle-mediated in-vivo base editing, a marked decrease in plasma ANGPTL3, triglycerides, and LDL-C was achieved [36^▪^]. This therapeutic effect was stable for at least 100 days after a single dose administration. These data highlight the potential of gene editing as an effective tool for correcting dyslipidemia and reducing cardiovascular risk.

### Vaccine

Although vaccination is mainly used as a prophylactic strategy for infectious diseases, there is growing interest in the use of vaccines for preventing noninfectious diseases, such as cardiovascular disease. In mice, vaccines targeting PCSK9 were shown to markedly lower plasma LDL-C levels [37]. In response, a number of phase 1 human clinical trials with PCSK9 vaccine have been completed or initiated [38]. With respect to ANGPTL3, a recent study showed that injection of a vaccine targeting the LPL-binding domain of ANGPTL3 significantly reduced fasting and postprandial plasma triglyceride levels in mice [39^▪^]. No effect of ANGPTL3 vaccination was observed on plasma cholesterol levels. Future studies should be conducted to determine to what extent ANGPTL3 vaccines may be able to lower plasma LDL-C.

## MECHANISM OF LDL-C LOWERING BY ANGPTL3 INACTIVATION

Although the stimulatory effect of ANGPTL3 on plasma triglycerides is unmistakably linked to inhibition of LPL, the mechanism underlying the decrease in LDL-C by ANGPTL3 deficiency or inactivation has remained a bit of a conundrum. Several mechanisms have been put forward for the LDL-C lowering effect of ANGPTL3 inactivation, some of which based on studies performed in ANGPTL3-deficient mice and others based on studies in mice or patients treated with monoclonal ANGPTL3 antibodies. Below, each of these potential mechanisms will be reviewed.

### VLDL secretion

Inactivation of ANGPTL3 using antibodies, antisense oligonucleotides, or gene ablation was found to be associated with decreased VLDL-TG or APOB secretion [29,30,40]. By contrast, other studies have not found a significant change in VLDL-TG secretion upon ANGPTL3 ablation or inactivation [7,8,41^▪^]. Although VLDL secretion may not be the primary target of ANGPTL3, based on the collective data, it is too early to fully dismiss decreased VLDL-TG and VLDL-APOB secretion as a mechanism contributing to LDL-C-lowering by ANGPTL3 inactivation.

### Lipoprotein lipase and lipoprotein receptors

Another possible explanation for the lowering of LDL-C by ANGPTL3 inactivation is that the increased LPL activity promotes hydrolysis of VLDL triglycerides, thereby enhancing clearance and/or reducing production of LDL precursors. One could wonder, though, why carrier status of loss-of-function variants in ANGPTL4, which is similarly associated with increased LPL activity and enhanced plasma triglyceride clearance, is not significantly associated with LDL-C [42]. With respect to lipoprotein receptors, studies have shown that the reduction in plasma LDL-C is not mediated by APOE, LDLR, SCARB1, CD36, LRP1 or SDC1, as LDL-C lowering by ANGPTL3 inactivation was maintained in mice lacking these proteins [41^▪^,^43^].

### Role of endothelial lipase

**FIGURE 2:**
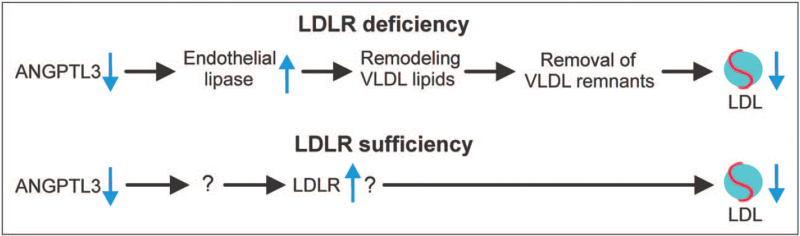
Mechanism by which ANGPTL3 inactivation reduces plasma LDL-C levels. In the absence of LDLR, ANGPTL3 inactivation reduces plasma LDL-C by promoting endothelial lipase-mediated processing of VLDL, leading to enhanced VLDL remnant clearance. In the presence of LDLR, ANGPTL3 inactivation reduces LDL-C likely by stimulating LDLR via a yet to be identified mechanism.

Two recent studies in mice suggest that in the absence of LDLR, the lowering of plasma LDL-C by ANGPTL3 deficiency is dependent on endothelial lipase, encoded by *Lipg*. Specifically, in mice in which *Ldlr* was silenced or ablated, genetic or immunological inactivation of ANGPTL3 decreased LDL-C in *Lipg*+/+ mice but not *Lipg*-/- mice [41^▪^,44^▪^]. By contrast, lowering of plasma triglycerides by ANGPTL3 inactivation was unaffected by *Lipg* genotype. Endothelial lipase thus appears to be a key mediator of the suppressive effect of ANGPTL3 inactivation on plasma LDL-C in the absence of LDLR (Fig. 2). Mechanistically, it was suggested that by derepressing endothelial lipase, ANGPTL3 inactivation causes remodeling of VLDL composition, resulting in the formation of lipid-depleted remnant particles [41^▪^]. These remnant particles are more rapidly cleared from the circulation, thereby depleting the LDL precursor pool and reducing LDL-C levels (Fig. 2). Consistent with this notion, changes in the lipidome of plasma lipoproteins were detected in ANGPTL3-deficient human subjects [45]. This proposed mechanism matches with data from four HoFH patients, showing that Evinacumab treatment is associated with a marked increase in fractional catabolic rate of IDL APOB [46]. Interestingly, Evinacumab treatment was also associated with an increase in fractional catabolic rate of LDL APOB, suggesting that ANGPTL3 inhibition may also directly affect LDL APOB metabolism in HoFH patients. How an enzyme, such as endothelial lipase, which only has a minor influence on LDL-C levels in the presence of LDLR, can account for the marked effect of ANGPTL3 inactivation on LDL-C in the absence of LDLR remains unexplained.

Intriguingly, the LDL-C-lowering effect of ANGPTL3 inactivation was independent of endothelial lipase in the presence of LDLR [41^▪^]. This makes sense because as indicated above, the impact of endothelial lipase on LDL-C levels is minor when LDLR is present. In line with the notion that ANGPTL3 inactivation does not enhance endothelial lipase activity when LDLR is present, carriers of loss-of-function variants in ANGPTL3 have similar HDL-C levels as noncarriers [21,22]. In the presence of LDLR, the mechanism for LDL-C lowering by ANGPTL3 inactivation likely involves LDLR, and may be characterized by enhanced LDL uptake [30]. Overall, ANGPTL3 inactivation may thus reduce LDL-C via two routes, one in the absence of LDLR via endothelial lipase and one independent of endothelial lipase via the LDLR (Fig. 2).

## FUTURE PERSPECTIVES AND CONCLUSION

With the recent approval of Evinacumab as treatment for HoFH, ANGPTL3 is fulfilling its promise as a target for lipid lowering, 20 years after its discovery. Apart from HoFH, there is major potential for targeting ANGPTL3 in the treatment of more common forms of dyslipidemia including HeFH, diabetic dyslipidemia, and refractory hypercholesterolemia. As is the case for therapies targeting PCSK9, if RNA-based therapies targeting ANGPTL3 turn out to be equally effective as antibody-based therapies, it can be expected that RNA-based approaches may ultimately prevail because of a lower frequency of injections. One of the major outstanding questions is whether inactivation of ANGPTL4 and/or ANGPTL8 on top of ANGPTL3 inactivation provides an additional therapeutic benefit. Inasmuch as whole body inactivation of ANGPTL4 was associated with mesenteric lymphadenopathy in mice and monkeys, targeting of ANGPTL4 should be liver-specific. Although ANGPTL3 and ANGPTL4 both inactivate LPL, they seem to operate independently and under different physiological circumstances, which implies that their concurrent inactivation may provide an additive benefit. Currently, however, there are no experimental data on the lipid-lowering effect of combined ANGPTL3/ANGPTL4 inactivation. Similarly, there are no data on the impact of combined ANGPTL3/ANGPTL8 inactivation on plasma triglycerides and LDL-C. Interestingly, mice deficient in both ANGPTL3 and ANGPTL8 were hypermetabolic and exhibited decreased fat mass [47], suggesting that combined ANGPTL3/ANGPTL8 inactivation may confer metabolic benefits beyond lowering of plasma lipids.

In conclusion, ANGPTL3 is an important liver-derived regulator of plasma lipoprotein metabolism. Clinical trials have demonstrated the potent lipid-lowering effects of ANGPTL3 inactivation. In the future, anti-ANGPTL3 therapies will be a valuable treatment option for reducing plasma LDL-C and triglycerides in selected patient groups.

## Acknowledgements

*None.*

### Financial support and sponsorship

*None.*

### Conflicts of interest

*There are no conflicts of interest.*

## REFERENCES AND RECOMMENDED READING

Papers of particular interest, published within the annual period of review, have been highlighted as:

▪ of special interest
▪▪ of outstanding interest

## References

[1] Mach F Baigent C Catapano AL. 2019 ESC/EAS Guidelines for the management of dyslipidaemias: lipid modification to reduce cardiovascular risk. Eur Heart J 2020; 41:111–188. 31504418 10.1093/eurheartj/ehz455

[2] Lotta LA Stewart ID Sharp SJ. Association of genetically enhanced lipoprotein lipase-mediated lipolysis and low-density lipoprotein cholesterol-lowering alleles with risk of coronary disease and type 2 diabetes. JAMA Cardiol 2018; 3:957–966. 30326043 10.1001/jamacardio.2018.2866 PMC6217943

[3] Stitziel NO Stirrups KE. Myocardial Infarction Genetics and CARDIoGRAM Exome Consortia Investigators. Coding variation in ANGPTL4, LPL, and SVEP1 and the risk of coronary disease. N Engl J Med 2016; 374:1134–1144. 26934567 10.1056/NEJMoa1507652 PMC4850838

[4] Ginsberg HN Packard CJ Chapman MJ. Triglyceride-rich lipoproteins and their remnants: Metabolic insights, role in atherosclerotic cardiovascular disease, and emerging therapeutic strategies. *Eur. Heart J* [in press]. 10.1093/eurheartj/ehab551 PMC8670783 34472586

[5] Kersten S. Physiological regulation of lipoprotein lipase. Biochim Biophys Acta 2014; 1841:919–933. 24721265 10.1016/j.bbalip.2014.03.013

[6] Koishi R Ando Y Ono M. Angptl3 regulates lipid metabolism in mice. Nat Genet 2002; 30:151–157. 11788823 10.1038/ng814

[7] Shimizugawa T Ono M Shimamura M. ANGPTL3 decreases very low density lipoprotein triglyceride clearance by inhibition of lipoprotein lipase. J Biol Chem 2002; 277:33742–33748. 12097324 10.1074/jbc.M203215200

[8] Ando Y Shimizugawa T Takeshita S. A decreased expression of angiopoietin-like 3 is protective against atherosclerosis in apoE-deficient mice. J Lipid Res 2003; 44:1216–1223. 12671033 10.1194/jlr.M300031-JLR200

[9] Fujimoto K Koishi R Shimizugawa T Ando Y. Angptl3-null mice show low plasma lipid concentrations by enhanced lipoprotein lipase activity. Exp Anim 2006; 55:27–34. 16508209 10.1538/expanim.55.27

[10] Shimamura M Matsuda M Yasumo H. Angiopoietin-like protein3 regulates plasma HDL cholesterol through suppression of endothelial lipase. Arterioscler Thromb Vasc Biol 2007; 27:366–372. 17110602 10.1161/01.ATV.0000252827.51626.89

[11] Ruppert PMM Michielsen C Hazebroek EJ. Fasting induces ANGPTL4 and reduces LPL activity in human adipose tissue. Molecular metabolism 2020; 40:101033. 32504883 10.1016/j.molmet.2020.101033 PMC7334813

[12] Quagliarini F Wang Y Kozlitina J. Atypical angiopoietin-like protein that regulates ANGPTL3. Proc Natl Acad Sci U S A 2012; 109:19751–19756. 23150577 10.1073/pnas.1217552109 PMC3511699

[13] Chi X Britt EC Shows HW. ANGPTL8 promotes the ability of ANGPTL3 to bind and inhibit lipoprotein lipase. Mol Metab 2017; 6:1137–1149. 29031715 10.1016/j.molmet.2017.06.014 PMC5641604

[14] Haller JF Mintah IJ Shihanian LM. ANGPTL8 requires ANGPTL3 to inhibit lipoprotein lipase and plasma triglyceride clearance. J Lipid Res 2017; 58:1166–1173. 28413163 10.1194/jlr.M075689 PMC5454515

[15▪▪] Chen YQ Pottanat TG Siegel RW. Angiopoietin-like protein 8 differentially regulates ANGPTL3 and ANGPTL4 during postprandial partitioning of fatty acids. J Lipid Res 2020; 61:1203–1220. 32487544 10.1194/jlr.RA120000781 PMC7397750

[16▪] Oldoni F Cheng H Banfi S. ANGPTL8 has both endocrine and autocrine effects on substrate utilization. JCI insight 2020; 5:e138777. 10.1172/jci.insight.138777 PMC7526440 32730227

[17▪▪] Kovrov O Kristensen KK Larsson E. On the mechanism of angiopoietin-like protein 8 for control of lipoprotein lipase activity. J Lipid Res 2019; 60:783–793. 30686789 10.1194/jlr.M088807 PMC6446706

[18] Chen YQ Pottanat TG Zhen EY. ApoA5 lowers triglyceride levels via suppression of ANGPTL3/8-mediated LPL inhibition. J Lipid Res 2021; 62:100068. 33762177 10.1016/j.jlr.2021.100068 PMC8079461

[19] Kathiresan S Melander O Guiducci C. Six new loci associated with blood low-density lipoprotein cholesterol, high-density lipoprotein cholesterol or triglycerides in humans. Nat Genet 2008; 40:189–197. 18193044 10.1038/ng.75 PMC2682493

[20] Musunuru K Pirruccello JP Do R. Exome sequencing, ANGPTL3 mutations, and familial combined hypolipidemia. N Engl J Med 2010; 363:2220–2227. 20942659 10.1056/NEJMoa1002926 PMC3008575

[21] Dewey FE Gusarova V Dunbar RL. Genetic and pharmacologic inactivation of ANGPTL3 and cardiovascular disease. N Engl J Med 2017; 377:211–221. 28538136 10.1056/NEJMoa1612790 PMC5800308

[22] Helgadottir A Gretarsdottir S Thorleifsson G. Variants with large effects on blood lipids and the role of cholesterol and triglycerides in coronary disease. Nat Genet 2016; 48:634–639. 27135400 10.1038/ng.3561 PMC9136713

[23] Gusarova V Alexa CA Wang Y. ANGPTL3 blockade with a human monoclonal antibody reduces plasma lipids in dyslipidemic mice and monkeys. J Lipid Res 2015; 56:1308–1317. 25964512 10.1194/jlr.M054890 PMC4479335

[24] Ahmad Z Banerjee P Hamon S. Inhibition of angiopoietin-like protein 3 with a monoclonal antibody reduces triglycerides in hypertriglyceridemia. Circulation 2019; 140:470–486. 31242752 10.1161/CIRCULATIONAHA.118.039107 PMC6686956

[25] Gaudet D Gipe DA Pordy R. ANGPTL3 inhibition in homozygous familial hypercholesterolemia. N Engl J Med 2017; 377:296–297. 28723334 10.1056/NEJMc1705994

[26▪▪] Raal FJ Rosenson RS Reeskamp LF. ELIPSE HoFH Investigators. Evinacumab for homozygous familial hypercholesterolemia. N Engl J Med 2020; 383:711–720. 32813947 10.1056/NEJMoa2004215

[27] Reeskamp LF Nurmohamed NS Bom MJ. Marked plaque regression in homozygous familial hypercholesterolemia. Atherosclerosis 2021; 327:13–17. 34004483 10.1016/j.atherosclerosis.2021.04.014

[28▪▪] Rosenson RS Burgess LJ Ebenbichler CF. Evinacumab in patients with refractory hypercholesterolemia. N Engl J Med 2020; 383:2307–2319. 33196153 10.1056/NEJMoa2031049

[29] Graham MJ Lee RG Brandt TA. Cardiovascular and metabolic effects of ANGPTL3 antisense oligonucleotides. N Engl J Med 2017; 377:222–232. 28538111 10.1056/NEJMoa1701329

[30] Xu YX Redon V Yu H. Role of angiopoietin-like 3 (ANGPTL3) in regulating plasma level of low-density lipoprotein cholesterol. Atherosclerosis 2018; 268:196–206. 29183623 10.1016/j.atherosclerosis.2017.08.031 PMC5750127

[31] Brandt TA Lee RG Digenio A. ISIS-ANGPTL3RX, an antisense inhibitor to angiopoietin-like 3, reduces plasma lipid levels in mouse models and in healthy human volunteers. Atherosclerosis 2015; 241:E30–E31.

[32▪▪] Gaudet D Karwatowska-Prokopczuk E Baum SJ. Vupanorsen, an N-acetyl galactosamine-conjugated antisense drug to ANGPTL3 mRNA, lowers triglycerides and atherogenic lipoproteins in patients with diabetes, hepatic steatosis, and hypertriglyceridaemia. Eur Heart J 2020; 41:3936–3945. 32860031 10.1093/eurheartj/ehaa689 PMC7750927

[33] Watts GF Schwabe R Scott R. RNA interference targeting hepatic angiopoietin-like protein 3 results in prolonged reductions in plasma triglycerides and LDL-C in human subjects. Circulation 2019; 140:e965–e1011.

[34] Watts GF Schwabe C Scott R. Abstract 15751: pharmacodynamic effect of ARO-ANG3, an investigational RNA interference targeting hepatic angiopoietin-like protein 3, in patients with hypercholesterolemia. Circulation 2020; 142:A15751–A115751.

[35] Chadwick AC Evitt NH Lv W Musunuru K. Reduced blood lipid levels with in vivo CRISPR-Cas9 base editing of ANGPTL3. Circulation 2018; 137:975–977. 29483174 10.1161/CIRCULATIONAHA.117.031335 PMC5830171

[36▪] Qiu M Glass Z Chen J. Lipid nanoparticle-mediated codelivery of Cas9 mRNA and single-guide RNA achieves liver-specific in vivo genome editing of Angptl3. Proc Natl Acad Sci U S A 2021; 118. 10.1073/pnas.2020401118 PMC7958351 33649229

[37] Toth S Pella D Fedacko J. Vaccines targeting PSCK9 for the treatment of hyperlipidemia. Cardiol Ther 2020; 9:323–332. 32737796 10.1007/s40119-020-00191-6 PMC7394273

[38] Zeitlinger M Bauer M Reindl-Schwaighofer R. A phase I study assessing the safety, tolerability, immunogenicity, and low-density lipoprotein cholesterol-lowering activity of immunotherapeutics targeting PCSK9. Eur J Clin Pharmacol 2021; 77:1473–1484. 33969434 10.1007/s00228-021-03149-2 PMC8440313

[39▪] Fowler A Sampson M Remaley AT Chackerian B. A VLP-based vaccine targeting ANGPTL3 lowers plasma triglycerides in mice. Vaccine 2021; 39:5780–5786. 34474934 10.1016/j.vaccine.2021.08.077 PMC8453115

[40] Wang Y Gusarova V Banfi S. Inactivation of ANGPTL3 reduces hepatic VLDL-triglyceride secretion. J Lipid Res 2015; 56:1296–1307. 25954050 10.1194/jlr.M054882 PMC4479334

[41▪] Adam RC Mintah IJ Alexa-Braun CA. Angiopoietin-like protein 3 governs LDL-cholesterol levels through endothelial lipase-dependent VLDL clearance. J Lipid Res 2020; 61:1271–1286. 32646941 10.1194/jlr.RA120000888 PMC7469887

[42] Dewey FE Gusarova V O’Dushlaine C. Inactivating variants in ANGPTL4 and risk of coronary artery disease. N Engl J Med 2016; 374:1123–1133. 26933753 10.1056/NEJMoa1510926 PMC4900689

[43] Wang Y McNutt MC Banfi S. Hepatic ANGPTL3 regulates adipose tissue energy homeostasis. Proc Natl Acad Sci U S A 2015; 112:11630–11635. 26305978 10.1073/pnas.1515374112 PMC4577179

[44▪] Wu L Soundarapandian MM Castoreno AB. LDL-cholesterol reduction by ANGPTL3 inhibition in mice is dependent on endothelial lipase. Circ Res 2020; 127:1112–1114. 32808882 10.1161/CIRCRESAHA.120.317128 PMC10150441

[45] Ruhanen H Haridas PAN Minicocci I. ANGPTL3 deficiency alters the lipid profile and metabolism of cultured hepatocytes and human lipoproteins. Biochim Biophys Acta Mol Cell Biol Lipids 2020; 1865:158679. 32151767 10.1016/j.bbalip.2020.158679

[46] Reeskamp LF Millar JS Wu L. ANGPTL3 inhibition with evinacumab results in faster clearance of IDL and LDL apoB in patients with homozygous familial hypercholesterolemia-brief report. Arterioscler Thromb Vasc Biol 2021; 41:1753–1759. 33691480 10.1161/ATVBAHA.120.315204 PMC8057526

[47] Banfi S Gusarova V Gromada J. Increased thermogenesis by a noncanonical pathway in ANGPTL3/8-deficient mice. Proc Natl Acad Sci U S A 2018; 115:E1249–E1258. 29358393 10.1073/pnas.1717420115 PMC5819435

